# Effects of Aging Treatment on the Microstructures and Mechanical Properties of a TC18 Alloy

**DOI:** 10.3390/ma17030570

**Published:** 2024-01-25

**Authors:** Song Zhang, Yong-Cheng Lin, Li-Hua Wang, Hong-Bo Ding, Yu-Liang Qiu

**Affiliations:** 1 Light Alloy Research Institute, Central South University, Changsha 410083, China; 2 School of Mechanical and Electrical Engineering, Central South University, Changsha 410083, China; 3 State Key Laboratory of Precision Manufacturing for Extreme Service Performance, Changsha 410083, China; 4 China Nonferrous Metals Processing Technology Co., Ltd., Luoyang 471039, China; 5 Rongcheng Huadong Metal-Forming Machinery Co., Ltd., Rongcheng 264300, China

**Keywords:** TC18 alloy, aging treatment, microstructures, mechanical properties, fracture mechanisms

## Abstract

In the present work, the effects of aging treatment on the microstructures of a TC18 alloy are studied. The influence of aging treatment on the tensile properties and failure mechanisms is systematically analyzed. It is found that the size and morphology of the primary α (α_p_) phases are insensitive to aging temperature and time. Furthermore, the aging temperature and time dramatically influence the precipitation of the secondary α (α_s_) phases. Massive α_s_ phases precipitate and gradually coarsen, and finally weave together by increasing the aging temperature or extending the aging time. The variations in α_p_ and α_s_ phases induced by aging parameters also affect the mechanical properties. Both yield strength (YS) and ultimate tensile strength (UTS) first increase and then decrease by increasing the aging temperature and time, while ductility first decreases and then increases. There is an excellent balance between the strengths and ductility. When the aging temperature is changed from 450 to 550 °C, YS varies from 1238.6 to 1381.6 MPa, UTS varies from 1363.2 to 1516.8 MPa, and the moderate elongation ranges from 9.0% to 10.3%. These results reveal that the thickness of α_s_ phases is responsible for material strengths, while the content of α phases can enhance material ductility. The ductile characteristics of the alloy with coarser α_s_ phases are more obvious than those with thinner α_s_ phases. Therefore, the aging treatment is helpful for the precipitation and homogeneous distribution of α_s_ phases, which are essential for balancing the strengths and ductility of the studied Ti alloy.

## 1. Introduction

Near-β-Ti alloys such as Ti-10V-2Fe-3Al, Ti-5Al-5V-5Mo-3Cr, and Ti-5Al-5Mo-5V-1Cr-1Fe (TC18) have been widely used in the aeronautical industry due to their excellent performance in strength, toughness, and corrosion resistance [1,2]. In general, the relationship between the desired microstructures and excellent mechanical properties of Ti alloys is inextricable, and the properties can be adjusted by tailoring the proportions or morphologies of α or β phases using heat treatment [3,4,5]. Hence, the diversity of microstructures and the complex influence of microstructure on properties in Ti alloys have stimulated ever-increasing research interests [6,7,8,9].

Usually, α phases are crucial to balance the mechanical properties, such as the strength and ductility of Ti alloys [10,11,12,13,14]. Duplex microstructures possess both good ductility and high strengths compared with other typical microstructures (equiaxed, widmannstatten, and basket-weave microstructures) [15,16,17,18,19]. The duplex microstructures can be induced by typical thermo-mechanical paths [20,21,22,23,24,25,26,27], in particular the aging conditions. Therefore, in recent decades, some studies have contributed to obtaining these considerable microstructures of Ti alloys by solution plus aging treatment [17,22,26]. Solution plus single aging was usually used by some researchers who found that the α_p_ phases are highly sensitive to the solution temperature, while the α_s_ phases are greatly affected by aging conditions [16,22]. Moreover, the precipitation of α_s_ phases could significantly enhance the properties of Ti alloys [28,29,30]. Shaha et al. [31] reported fine α phases and low residual stress in the Ti55511 alloy, resulting in improved mechanical properties. Qiu [32] provided a rapid heat treatment (RHT) and subsequent aging for a Ti55511 alloy and avoided the formation of the ω phase, which results in refined α_s_ precipitations. Also, the uniform and ultra-fine α_s_ phases were obtained by solution plus duplex aging [33]. Liu et al. [34] also reported a two-step aging treatment for a Ti55511 alloy and obtained the uniformly distributed α_p_ and α_s_ phases. In recent years, multi-stage treatment methods have been employed to optimize the mechanical properties of Ti alloys [35]. It is indicated that the content of the α_p_ phase is relatively stable after the multi-stage treatment, while the size of the α_s_ phase becomes non-uniform and the content of the α_s_ phase increases with an increasing cooling rate. Additionally, the influence of aged microstructures on mechanical properties was investigated. Guo et al. [16] demonstrated that the strength of the damage-tolerant Ti-6Al-4V (TC4-DT) alloy with duplex microstructures is minimally impacted by the α_p_ phase, while ductility significantly increases as the content of the α_p_ phase increases. In other words, α_s_ phases are vital for improving strength and ductility. Also, the α_p_ phase can improve the fracture toughness by changing the crack propagation paths and augmenting energy consumption [36]. Shi et al. [37] found that the presence of weaved lamellar α phases is beneficial for improving the fracture toughness of a Ti-55511 alloy. Aeby-Gautier et al. [38] examined that the formation of the α_s_ phase during aging can increase tensile strength. Hence, the tailoring of α_p_ and α_s_ phases is the key to obtaining excellent properties of Ti alloys with duplex microstructures.

Nevertheless, previous researchers have devoted themselves to elaborating on the influence of heating paths on the morphologies and properties of phases. But, less attention was paid to the interactions between heat treatment, phases, mechanical properties, and the quantitative characterization of α_p_ and α_s_ phases in aged near-β-Ti alloys. Furthermore, the relationship between the content/size of phase and tensile properties is also inexplicit. The quantitative relation has also not been directly testified by experimental evidence under different aging conditions. Especially, the evolution law of α_s_ phases and mechanical properties under different aging conditions are less involved. Therefore, the influences of aging conditions on the content and morphologies of α_p_ and α_s_ phases in a near-β-Ti alloy (TC18) are analyzed. The effects of crucial microstructure features on mechanical properties are also discussed. Subsequently, the tensile failure mechanisms are revealed. These experimental findings and understandings are useful for improving the performance of the TC18 alloy.

## 2. Material and Experiment Procedures

**Figure 1:**
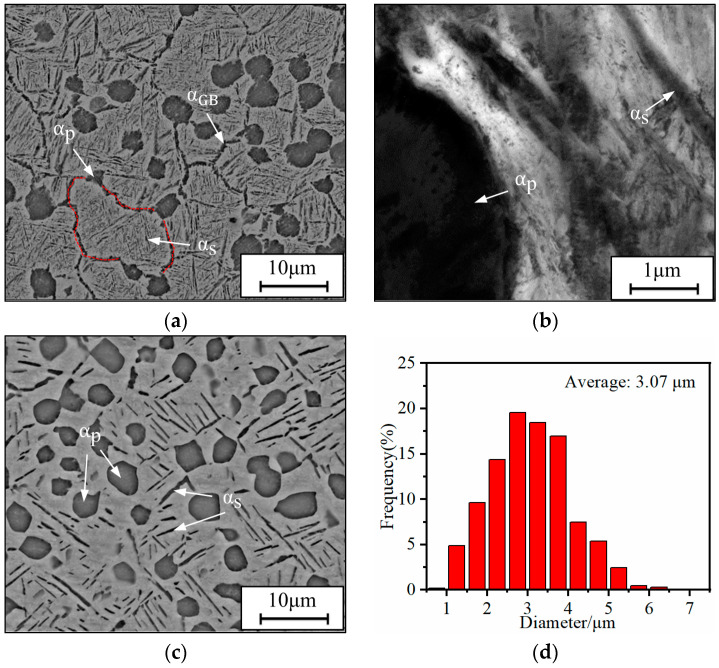
Microstructures of the as-received alloy: (**a**) SEM observation; (**b**) TEM observation (bright field); (**c**) SEM observation (pretreated); (**d**) particle size distribution of α_p_ phases (pretreated).

The chemical compositions (wt.%) of the studied TC18 alloy are presented as follows: 5.16 Al, 4.92 Mo, 4.94 V, 1.10 Cr, 0.98 Fe, and balance Ti. The transition temperature of the full β phase is 875 ± 5 °C [1,5]. The original microstructures are presented in Figure 1a,b. There are some discontinuous grain boundary α phases (α_GB_), α_p_ phases, α_s_ phases, and a β matrix (β_m_). The solid solution combined with aging treatment remains a crucial approach for enhancing the mechanical properties of Ti alloys. According to the current national standards of the People’s Republic of China (GB/T 37584-2019 [39]), the temperature and time parameters are specified for the heat treatment process of the studied alloy. Specifically, for the solution treatment, the recommended upper limit of temperature is 780 °C at a duration is 3 h. Similarly, for the aging treatment, the recommended upper limit is 600 °C for 10 h. Consequently, all specimens were pretreated at 780 °C for 1 h and then cooled by water quenching (WQ) before aging. The pretreated microstructure is displayed in Figure 1c, and Figure 1d shows the size distribution of α_p_ phases. Several aging treatment paths were designed, and an air cooling (AC) method was performed after aging treatment, as presented in Table 1.

The specimens for metallographic examination were cut into slices using electrical discharge machining. The section of each specimen was prepared using standard metallurgical procedures. The specimens were first abraded by SiC paper from grid 400# to 2000# and polished with Al_2_O_3_ suspension liquid and then etched in a solution mixed with 2% HF, 5% HNO_3_, and 93% H_2_O [5]. In order to carry out transmission electron microscopy (TEM) observation, the thin foils were first ground by SiC paper and polished using twin jet electrochemical polishing in a solution of 5% perchloric acid, 35% normal butanol, and 60% methanol. The microstructure and fracture morphologies were characterized by the scanning electron microscopy (SEM) (FEI Electron Optics B.V; Prague, Czech Republic). The TEM (Tecnai G2 F20, FEI Company, Hillsboro, OR, USA) figures were captured by a Tecnai G2-20 microscope. Subsequently, Image-pro plus 6.0 software was employed to measure the content, grain diameters, and thickness of the phases based on three images from distinct areas. Tensile specimens with a gauge length of 30 mm and a diameter of 6 mm were prepared and then polished with 400-1000# grid SiC paper. The tensile tests were performed in an MTS-GWT2105 test machine (MTS System Corporation, Eden Prairie, MN, USA). With a tensile rate of 1.8 mm/min, three samples were tested under each tested condition. The reported results represent the average values obtained from these replicate tests.

## 3. Results and Discussion

### 3.1. Effects of Aging Conditions on Microstructures

#### 3.1.1. Effects of Aging Temperature

**Figure 2:**
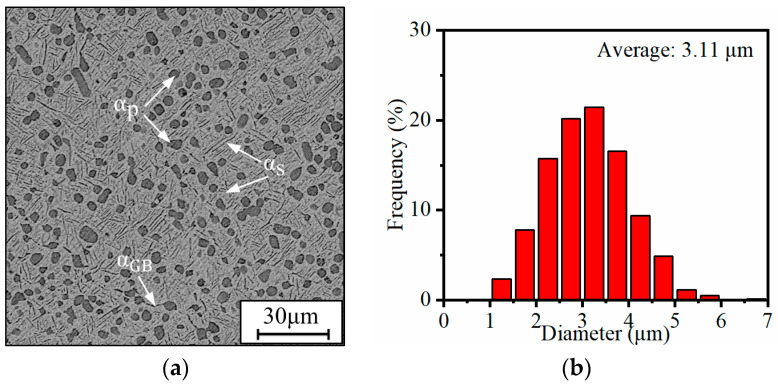

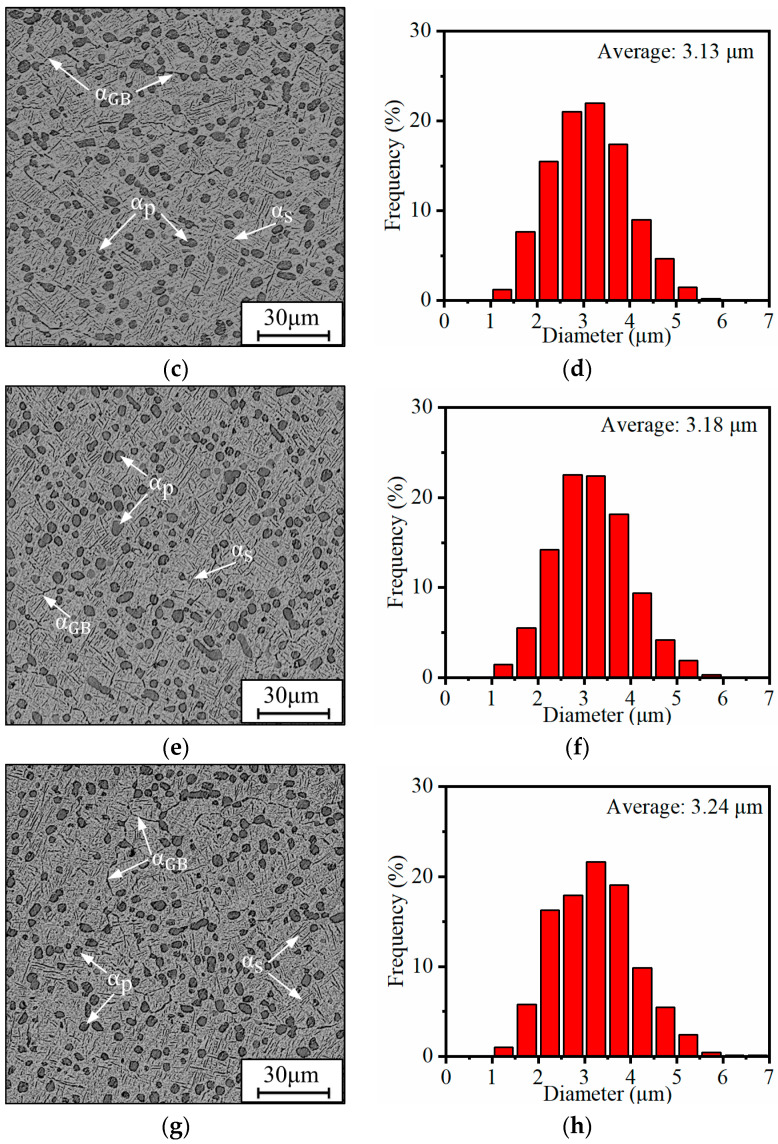
Microstructures and the size distribution of α_p_ phases of the TC18 alloy aged at (**a**,**b**) 450 °C; (**c**,**d**) 500 °C; (**e**,**f**) 550 °C; (**g**,**h**) 600 °C.

Figure 2 illustrates the influence of aging temperature on the microstructures and the size distribution of α_p_ phases. The α_p_ phases mainly distribute at β grain boundaries, and the size of the β grain is only a few tens of microns. This indicates that there is a pinning effect induced by α_p_ phases on the growth of the β grain during aging treatment (Figure 2a,c,e,g). By increasing the temperature, the size uniformity of α_p_ phase gradually decreases but the average size slightly increases. The average sizes of the α_p_ phase have been estimated to be 3.11 μm, 3.13 μm, 3.18 μm, and 3.24 μm (Figure 2b,d,f,h), respectively, when the alloy was aged at 450 °C, 500 °C, 550 °C, and 600 °C. The average size of the α_p_ phase is less than 3.3 μm, and the growth of the α_p_ phase is restricted during aging treatment. The morphology, size, and volume fraction of α_p_ phases exhibit overall stability or slight variations with the aging temperature (Table 2). This trend aligns with observations commonly reported in the literature [30]. There are two potential reasons. First, the onset temperature for the transformation from α phase to β phase in α + β titanium alloys is typically around 700 °C [30]. Given that the aging temperature is lower than this threshold, there is no transformation from the α phase to β phase. Second, the metastable β phase, following the solid solution, undergoes solute redistribution in the aging process. This leads to the precipitation of the metastable ω phase, facilitating the formation of the α_s_ phase. The morphology of the precipitated α_s_ phase is primarily lamellar, exhibiting a distinct difference from the morphology of the α_p_ phase. In the aging process, the transformation of metastable β phases to α phases is dominated by element diffusion. Therefore, the precipitation of α_s_ phases is slow, owing to the low driving force at lower temperatures. The α_s_ phases precipitate not only at the α/β interface but also within β_m_. The precipitation is dominated by increasing the diffusion driving force as the temperature is increased [40]. In summary, the effects of temperature on α_p_ phases are negligible because the metastable β phases mainly transform into α_s_ phases during the aging treatment.

**Figure 3:**
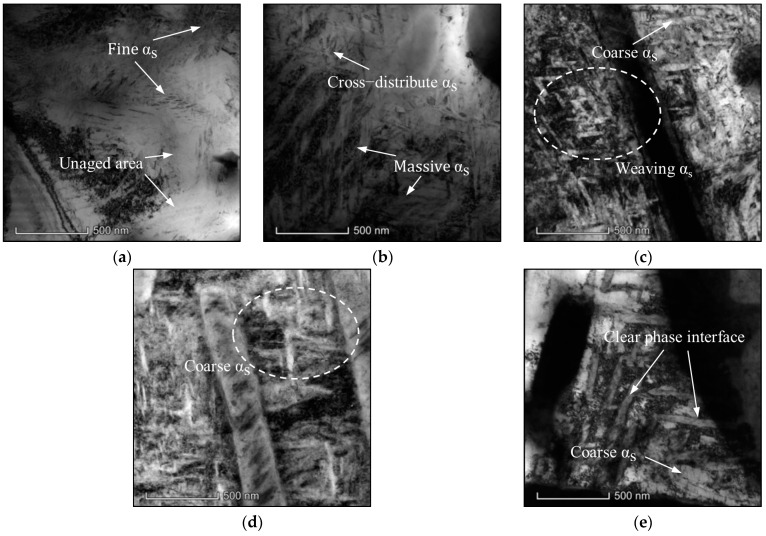
TEM micrographs of the TC18 alloy aged at (**a**) 400 °C; (**b**) 450 °C; (**c**) 500 °C; (**d**) 550 °C; (**e**) 600 °C.

Figure 3 shows the influence of aging temperature on the formation of α_s_ phases. A few dislocations and substructures are found. The precipitated α_s_ phases are cross-distributed irregularly, and their thickness varies from 0.2 to 0.6 μm. Also, the orientations of the precipitated α_s_ phases are random [41]. As illustrated in Figure 3a, α_s_ phases with a fine acicular shape precipitate, and several unaged areas appear in the β_m_ at an aging temperature of 400 °C. Meanwhile, distinguishing α_s_ phases from β_m_ is tricky because the boundaries of α_s_ phases are blurred. Due to the high content of β-stable elements in β_m_ and a smaller driving force for solute atom diffusion at lower aging temperatures, the transformation for β_m_ to α_s_ phases is insufficient (Figure 3a). Therefore, the content of α_s_ phases is extremely low, and the average thickness is small at lower aging temperatures. As the temperature increases, the massive α_s_ phases precipitate in a complete aging process (Figure 3b–d). Moreover, the α_s_ phases coarsen and tightly weave together. The α_s_/β interfaces are visible, especially at high aging temperatures, such as 600 °C, and the average thickness of α_s_ phases increases to about 0.47 μm (Table 2). So, α_s_ phases are sensitive to the variations in aging temperature, and the coarsening rate of α_s_ phases is obviously affected by the diffusivity of solute atoms [42].

#### 3.1.2. Effects of Aging Time

**Figure 4:**
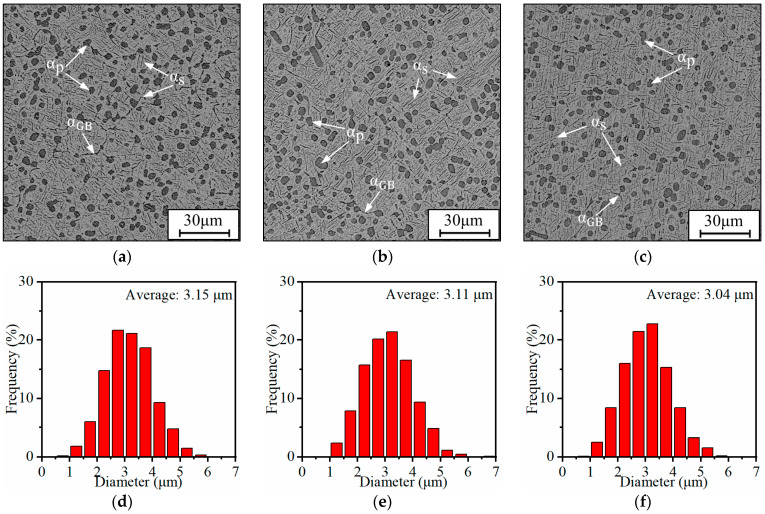
Microstructures and size distributions of α_p_ phases after being aged at 450 °C and the aging times of (**a**,**d**) 1 h; (**b**,**e**) 4 h; (**c**,**f**) 8 h.

Figure 4 illustrates the influence of aging time on the microstructures and the particle size distribution of the α_p_ phase when the alloy was aged at 450 °C. As shown in Figure 4a–c, there are spherical α phases, α_GB_, and β transition phases. By prolonging the aging time, the variations of α_p_ phases are inconspicuous. The particle size distribution and average size of α_p_ phases at different aging times are shown in Figure 4d–f. The average size and content of α_p_ phases fluctuate only slightly by increasing time, and the average sizes are 3.15, 3.11, and 3.04 μm, respectively, when the aging times are 1, 4, and 8 h. The content of α_p_ phases shows an opposite pattern, which first decreases and then increases, and the corresponding contents of α_p_ phases are 16.35%, 18.34%, and 16.53%, respectively.

**Figure 5:**
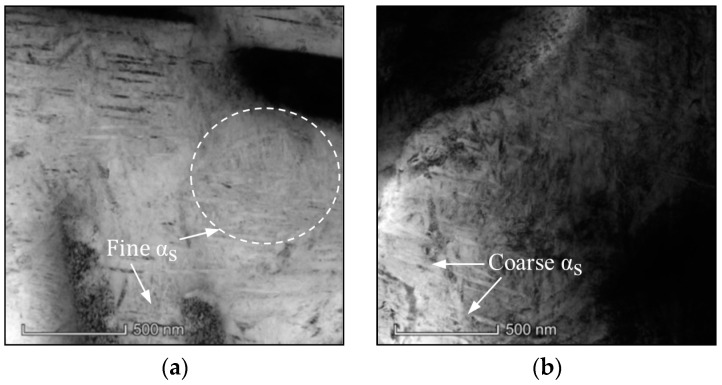

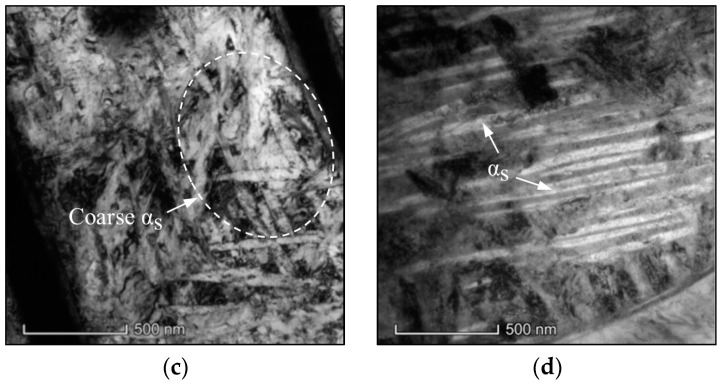
TEM micrographs of the TC18 alloy aged at (**a**) 450 °C/2 h/AC; (**b**) 450 °C/4 h/AC; (**c**) 500 °C/4 h/AC; (**d**) 500 °C/8 h/AC.

Figure 5 shows the influence of aging time on the α_s_ phases at different aging temperatures. At 450 °C, the fine acicular α_s_ phases appear in β_m_ when aged for 2 h (Figure 5a). When the aging time is 4 h, the morphologies of α_s_ phases change significantly and display a typical coarsening behavior (Figure 5b). At 500 °C, the same experimental results are obtained by increasing the aging time (Figure 5c,d). At both 450 °C and 500 °C, α_s_ phases become coarser, and the spaces between adjacent α_s_ phases become narrow by increasing the aging time. Actually, the thickness and interlamellar space of α_s_ phases are a little varied compared with the results shown in Figure 3. So, it can be concluded that the influence of aging time on the microstructures is weaker than aging temperature.

### 3.2. Effects of Aging Conditions on Tensile Properties

#### 3.2.1. Tensile Properties of the Aged TC18 Alloy

Table 3 gives the tensile properties of the TC18 alloy aged at different aging conditions. It is apparent that both aging temperature and time are responsible for the strength and ductility of the aged alloy. Compared with the alloy without aging treatment, the strength or ductility of the aged alloy has been improved. As the aging temperature increases, the strength increases first and then decreases. An excellent balance exists between the strength and ductility of the alloy aged at 450–550 °C. The yield strength (YS) ranges from 1238.6 to 1381.6 MPa, and the ultimate tensile strength (UTS) ranges from 1363.2 to 1516.8 MPa. Also, the moderate elongation (δ) ranges from 9.0% to 10.3%. When aged at 600 °C, UTS and YS are almost equal to those at 400 °C, while ductility is obviously different. There is a sharp increase in ductility (~15.1%). Yin et al. [43] reported that the highest acceptable YS and UTS are 1023 and 1122 MPa in an aged TC18 alloy, respectively, with an excellent ductility of 13.8%. They asserted that the excellent ductility is attributed to the large-size and high-volume fraction of the α_p_ phase. Recently, Li et al. [44] obtained a high YS and UTS of 1456 MPa and 1504 MPa, respectively, in a TC18 alloy. However, this improvement in strength is accompanied by a poor ductility of 1.5%. The higher strength and poor ductility can be ascribed to the smaller interlamellar spacing between α_s_ phases following a three-step heat treatment approach. Indeed, it is evident that the trade-off for excellent strength is poor ductility. Unlike common understandings, only a 0.9% loss of UTS (~12 MPa) can result in a ~34% increase in the elongation of the alloy. To further reveal the influences of aging time on the properties, the specimens were aged at 450 °C for 2, 4, and 8 h. UTS and YS are still relatively high (1263.4–1516.9 MPa), while ductility shows a concave parabolic tendency. In addition, the influence of aging temperature on tensile properties is more obvious than aging time, which is consistent with the analyses in Section 3.1. So, the α_s_ phases induced by aging treatment can optimize the properties of the TC18 alloy.

#### 3.2.2. Effects of Microstructural Features on the Tensile Properties of the Aged TC18 Alloy

The experimental findings above reveal the effects of aging conditions on microstructures, which result in the difference in properties. In general, the morphologies of α_s_ phases mainly determine the strengths, and the content of the α_p_ phase and the size of the β grain mainly control the ductility of near-β-Ti alloys with duplex microstructures [45,46,47]. Due to the specimens being pretreated by the same method in this work, the size of the β grain is insignificant in discussing the factors affecting the mechanical properties. As a rule, the strength of the near-β-Ti alloy increases by decreasing the aging temperature and time after the solution within the α + β region [28,41,46]. The trade-off for excellent strength is poor ductility. In fact, some differences with the aforementioned standpoints are presented in this work, e.g., both the strengths and ductility decrease (aged at 450 and 500 °C for 4 h). Such slightly contradictory phenomena might be a consequence of the competitive effects of the content of the α phase and the thickness of the α_s_ phase, i.e., the content of the α phase dramatically decreases (27.61–23.79%) while the thickness of the α_s_ phase slightly increases (0.30 to 0.35 μm, as shown in Table 2). The detailed changing trend of feature parameters is summarized in Table 4. The tensile strengths and ductility at an aging temperature of 500 °C are slightly reduced compared to those at 450 °C. Furthermore, the tensile strength continually decreases by increasing the content of the α phase or the thickness of the α_s_ phase when the aging temperature exceeds 500 °C (Table 4).

As described above, the fine acicular α_s_ phases precipitate in the β_m_ during aging treatment at low temperatures, such as 400 and 450 °C. These acicular α_s_ phases can create massive phase boundaries that act as dislocation barriers and effectively block the dislocations at interfaces. Thus, the strength increases. Nevertheless, the strength of the alloy aged at 450 °C is better than 400 °C. In fact, although the size of precipitated α_s_ phases is extremely fine when aged at 400 °C, its content is low due to the lower diffusion driving force. Therefore, the strength is lower than 450 °C. It must be mentioned that α_s_ phases become coarse by increasing the temperature and time. These coarse α_s_ phases increase the slip distance of dislocations and decrease the number of α/β phase boundaries. So, the strength decreases with increasing temperature and time. As indicated in the other report [48], the cavities are formed at the α/β phase interfaces during plastic deformation. Moreover, the cavities grow along with the α/β interfaces and coarsen or gather to form microcracks. The free path of the cavities grew, and the microcracks were hindered by α phases. Therefore, the more α phases there are, the shorter the free path is. These cavities encounter more obstacles in the growing progress [16], which improves ductility. In addition, the dislocations are activated at α/β interfaces and first slip in α phases with a lower resolved shear stress [38]. An increasing content in the α phase can increase the degree of uniform plastic deformation and thus improve ductility. Also, the mean-free path of dislocations (or slip) increases by increasing the sizes of α_s_ phases [22]. Especially at 600 °C, the content of the α phase tends to be stable (~26%), but ductility still is better than at 550 °C. This is a result of the coarsening and homogenization of the α_s_ phase. Therefore, the coarse α_s_ phases induced by increasing the temperature and time can contribute to the improvement of ductility. In short, the thickness of the α_s_ phase is responsible for strength, while the content of the α phase can enhance ductility.

#### 3.2.3. Crucial Roles Affecting Tensile Strengths of the TC18 Alloy

The properties of Ti alloys are bound with the microstructural features [35,49,50], e.g., the content of the α phase, the size of the β grain, the diameter of the α_p_ grain and the thickness of the α_s_ phase. In this work, the classical Hall–Petch formula is employed to express YS or UTS [41,51]:

(1) $$\sigma_{i}=\sigma_{s}+k_{i}^{A}v_{\beta }d_{\beta }^{-1/2}+k_{i}^{B}v_{\alpha_{p}}d_{\alpha_{p}}^{-1/2}+k_{i}^{C}v_{\alpha_{s}}l_{\alpha_{s}}^{-1/2}\left(i=\mathrm{YS},\;\mathrm{UTS}\right)$$

where $\sigma_{i}$ are the strengths (YS or UTS), $\sigma_{s}$ represents the total resistance determined by the crystalline structure and the dislocation density, and $k_{i}^{A}$, $k_{i}^{B}$, and $k_{i}^{C}$ are the pin constant of the β grain and α_p_ and α_s_ phases, respectively. *v* denotes the content or volume fraction of the β grain and α_p_ or α_s_ phases, *d* represents the diameter of the grain, and *l* is the thickness of the lamellar α_s_ phase.

In the present work, all specimens undergo the same solution treatment. Synthesizing the previous analysis, the involved eigenvalue of β grains and α_p_ phases are regarded as constants. So, $\sigma_{i}$ can be rewritten as:

(2) $$\sigma_{i}=f\left(v_{\alpha_{s}}l_{\alpha_{s}}^{-1/2}\right)+C$$

where *f* is a linear function of the eigenvalues $v_{\alpha_{s}}$ and $l_{\alpha_{s}}^{-1/2}$, and C represents a constant.

**Figure 6:**
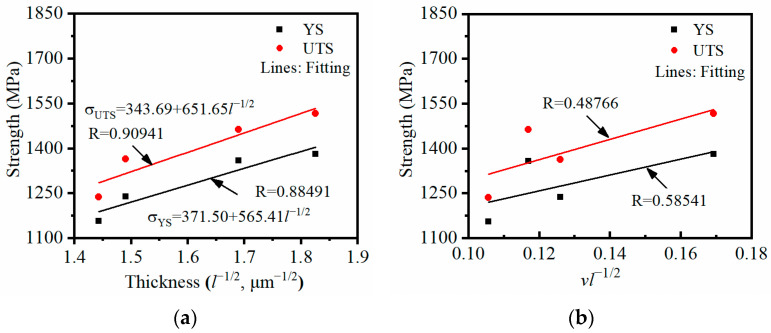
The relationship between the strengths and key factors involving α_s_ phases: (**a**) $\;\sigma_{i}$−$l_{\alpha_{s}}^{-1/2}$; (**b**) $\sigma_{i}$−$\;v_{\alpha_{s}}l_{\alpha_{s}}^{-1/2}$.

In Equation (2), it is apparently manifest that the suitable approach for improving the strength is increasing the volume fraction of the α_s_ phase or decreasing the thickness of the α_s_ phase. Figure 6 depicts the relationship between the strengths and key factors involving α_s_ phases. When the volume fraction of the α_s_ phase is not considered, a good linear correlation exists between the strengths and the reciprocal square root of the thickness of the α_s_ phase (Figure 6a). Meanwhile, a non-linear relation exists between the strengths, the product of $v_{\alpha_{s}}$, and the reciprocal square root of the α_s_ thickness *l* (Figure 6b). The results indicate that decreasing the thickness of the α_s_ phase can improve the strength, and the thickness of the α_s_ phase is a key factor affecting the strength of the TC18 alloy with duplex microstructures. The conclusions are also consistent with the experimental findings of Mora [47].

In summary, the thin α_s_ phases with large aspect ratios precipitate at low aging temperatures, which results in a high interface energy and external energy for plastic deformation [41]. The tensile strengths are excellent, and poor ductility appears. The α_s_ phases are coarse and shorter, which reduce the required external energy for plastic deformation. Thus, strength decreases and ductility increases at high aging temperatures.

### 3.3. Fractographies and Fracture Mechanisms

#### 3.3.1. Fractographies of TC18 Alloy

**Figure 7:**
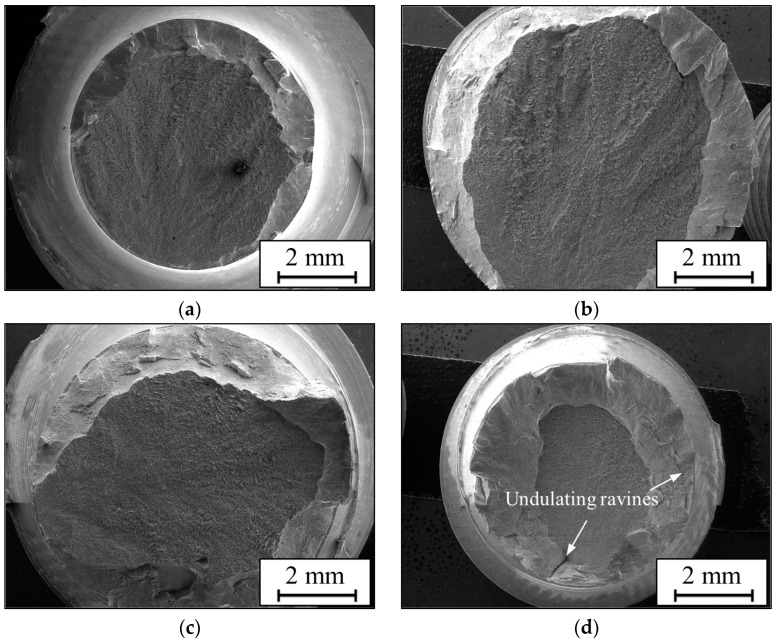
The macroscopic fracture of the TC18 alloy aged at (**a**) 450 °C; (**b**) 500 °C; (**c**) 550 °C; (**d**) 600 °C.

**Figure 8:**
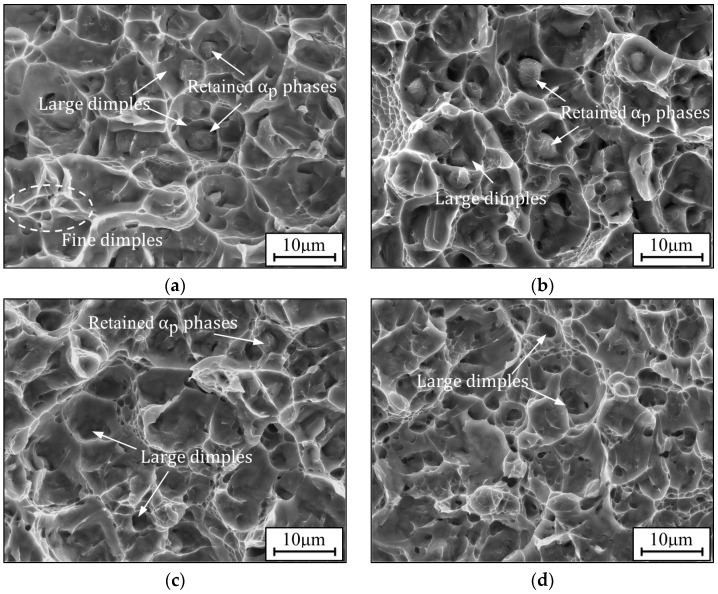
The microscopic fracture morphologies of the TC18 alloy aged at (**a**) 450 °C; (**b**) 500 °C; (**c**) 550 °C; (**d**) 600 °C.

The macroscopic fractures of the alloy at aging temperatures from 450 to 600 °C are illustrated in Figure 7. It can be noted that the surface roughness of fractures is quite different at the four aging paths. The surface morphologies vary from flatness to roughness by increasing the temperature. In particular, there are large undulating ravines on the fracture surface at 600 °C (Figure 7d), which indicate excellent ductility (~15.1%). The tensile fracture surfaces can be divided into two distinct zones: the fibrous and shear lip zone [52,53]. Figure 8 shows the microscopic images of the fibrous zone. Fracture surfaces under different aging conditions contain some dimples, but their sizes are unhomogeneous. The diameters of unhomogeneous irregular dimples are approximately 0.2–6.0 μm. Furthermore, a great deal of tear ridges and some facets gather around dimples. The proportion of unhomogeneous dimples is lower at lower temperatures (Figure 8a,b) but increases at higher temperatures (Figure 8c,d). Moreover, these unhomogeneous dimples in Figure 8a,b are shallower than those in Figure 8c,d.

#### 3.3.2. Fracture Mechanisms

Usually, the fracture behavior of the TC18 alloy is inextricable to microstructures. Tensile fracture failures of the alloy aged at different conditions are mixed mode, including quasi-cleavage and ductile failure. However, the fracture surface roughness and morphologies among the four aging routes are quite different. In addition, the degree of necking also varies dramatically. The fracture surface at 450 °C is flat (Figure 7a), and the surfaces become rougher by increasing the average thickness of the α_s_ phase (Figure 7c,d). A lot of unhomogeneous cavities that nucleate and coarsen along with the interface of the α phase and β_m_ induce the fracture failure of the studied alloy [54]. References [52,55] indicated that the interfaces of the α_p_/β_m_ phase are easy and can be the potential nucleation site of micro-voids due to the difficulty in coordinating deformation. The bottom sizes of these large dimples are approximately equal to the diameter of α_p_ phases (~3 μm). Some equiaxed α_p_ phases are retained at the bottom of dimples after fracture (Figure 8a–c). Although the retained α_p_ phases are not visible in Figure 8d, these large dimples are still induced by nucleation and coarsening at the α_p_/β_m_ interfaces. Moreover, some fine dimples exist around the large dimples (Figure 8a), and the size of the fine dimple is approximately equal to the thickness of the α_s_ phase. Because of the small thickness, α_s_ phases hardly accommodate plastic deformation. Thus, there are fewer dimples left after fracture (Figure 8a,b). However, the sizes of dimples induced by α_s_ phases gradually increase with increasing temperature (Figure 8c,d), which indicates that the coarsening of α_s_ phases makes a certain contribution to enhancing ductility. The data shown in Table 3 also verify this conclusion. In addition, α_s_ phases weave tightly in β_m_ by increasing temperature or time (Figure 3 and Figure 5), and the crack cannot avoid encountering coarse α_s_ phases during propagation. Thus, a larger plastic deformation zone will form nearer the crack tip of coarse α_s_ phases than those with no α_s_ phases or finer α_s_ phases in β_m_ [36,41]. All aforementioned consequences result in the phenomenon that the ductile failure characters of the alloy with coarser α_s_ phases are more obvious than those with thinner α_s_ phases. Therefore, it is feasible to enhance the ductility of the TC18 alloy by controlling the proper aging conditions to coarsen α_s_ phases.

## 4. Conclusions

The influences of aging conditions on the microstructures and properties of the TC18 alloy were systematically investigated. The crucial conclusions are shown below.

1. α_p_ phases are insensitive to the aging temperature and time. However, the temperature and time significantly affect α_s_ phases. The fine acicular α_s_ phases precipitate in β_m_ during aging treatments and become coarsened by increasing temperature or time.
2. The thickness of α_s_ phases is responsible for strength, while the content of α phases can enhance ductility. Nevertheless, an excellent balance exists between strength and ductility, particularly when aged at 450–550 °C. YS ranges from 1238.6 to 1381.6 MPa, UTS ranges from 1363.2 to 1516.8 MPa, and moderate elongations range from 9.0% to 10.3%.
3. The failures of the alloy aged under different conditions are mixed mode, including quasi-cleavage and ductile failure. The ductile fracture characteristics of the alloy with coarser α_s_ phases are more obvious than those with thinner α_s_ phases. Consequently, the coarsening of α_s_ phases makes a certain contribution to improving the ductility of the TC18 alloy with duplex microstructures.

## Figures and Tables

**Table 1:** Experimental schemes for heat treatment.

Route No.	Pretreatment	Aging Condition		
		Temperature (°C)	Hold Time (h)	Cooling Method
1	780 °C/1 h/WQ	400	4	AC
2		450		
3		500		
4		550		
5		600		
2–1		450	1	
2–2		450	2	
2–3		450	8	

**Table 2:** Content and size of phases with different aging conditions.

Aging Condition ^a^	The Content of α Phases (%)	The Content of α_p_ Phases (%)	The Diameter of α_p_ Phases (μm)	The Content of α_s_ Phases (%)	The Thickness of α_s_ Phases (μm)
450 °C	27.61 ± 2.12	18.34 ± 0.82	3.11 ± 0.09	9.27 ± 0.58	0.30 ± 0.027
500 °C	23.79 ± 2.78	16.87 ± 1.17	3.13 ± 0.07	6.92 ± 0.87	0.35 ± 0.016
550 °C	27.01 ± 1.05	18.56 ± 1.03	3.18 ± 0.14	8.45 ± 0.69	0.45 ± 0.021
600 °C	25.89 ± 1.53	18.57 ± 0.69	3.24 ± 0.12	7.32 ± 0.53	0.47 ± 0.017

^a^ Aging time of 4 h and subsequent AC.

**Table 3:** Tensile properties of the alloy aged for different conditions.

Pretreatment	Aging Conditions	YS/MPa	UTS/MPa	Elongation (δ)/%
780 °C/1 h/WQ	/	1220.2	1359.9	9.1
	400 °C/4 h/AC	1157.9	1248.2	11.3
	450 °C/2 h/AC	1263.4	1406.9	12.5
	450 °C/4 h/AC	1381.6	1516.8	9.3
	450 °C/8 h/AC	1353.7	1516.9	11.2
	500 °C/4 h/AC	1358.3	1463.5	9.0
	550 °C/4 h/AC	1238.6	1363.2	10.3
	600 °C/4 h/AC	1156.8	1236.7	15.1

**Table 4:** The detailed changing trend of feature parameters.

Temperature	The Content of α Phases	The Thickness of α_s_ Phases	The Tensile Strength	Ductility
450→500 °C	↓	↑	↓	↓
500→550 °C	↑	↑	↓	↑
550→600 °C	↓	↑	↓	↑

Symbols “↑” and “↓” represent an increase and decrease, respectively.

## Data Availability

The raw/processed data required to reproduce these findings cannot be shared at this time as the data also form part of an ongoing study.
